# Prostatic Abscess in a 45-Year-Old Male: Atypical Presentation and Successful Management With Open Transperineal Drainage

**DOI:** 10.7759/cureus.95656

**Published:** 2025-10-29

**Authors:** Nikita Rana, Ashwini Prakash

**Affiliations:** 1 Urology, Wexham Park Hospital, Slough, GBR; 2 Oncology, Royal Berkshire Hospital, Reading, GBR

**Keywords:** atypical, case report, general surgery, incision and drainage, prostate abscess, sepsis, transperineal open surgery, urological emergency, urology

## Abstract

A prostatic abscess is a rare but serious urological emergency that requires prompt recognition and intervention due to associated risks of urosepsis, multi-organ failure, and death. This case describes a previously healthy 45-year-old male who experienced delayed diagnosis and management of a complex prostatic abscess following multiple encounters with secondary care services. Imaging revealed a prostatic abscess with atypical features, including multiloculation and extension into the ischiorectal fossa and gluteal region. Given the extensive nature of the infection and preoperative signs of sepsis, emergency drainage was performed through an unconventional transperineal open approach in a joint procedure between Urology and General Surgery. This method was selected because aspiration alone, although considered the first-line approach for prostatic abscess drainage, was unlikely to provide adequate drainage given the multiloculated and extensive nature of the abscess. As no underlying cause for abscess formation was identified during inpatient admission, the patient underwent a comprehensive outpatient evaluation following acute management. This follow-up included detailed biochemical and clinical assessments, coupled with thorough history-taking, to identify any missed risk factors. This case underscores several key considerations for clinical practice, including the importance of early imaging to guide management, guideline-directed antibiotic therapies, robust follow-up and safety-netting systems, and timely multidisciplinary collaboration. It also demonstrates that an open transperineal drainage approach serves as a safe and effective treatment for atypical abscesses when conventional transurethral, transrectal, or minimally invasive transperineal methods are likely to be inadequate.

## Introduction

Prostate abscesses are characterized by the accumulation of purulent matter originating from within the prostate gland; they are typically walled off by a membrane of inflammatory tissue [1]. Although rare, they account for approximately 0.5% of all urological diseases and carry a mortality of up to 16% [1]. Typical prostate abscesses are localized, presenting as a single, simple fluid collection confined to the gland [1,2]. Atypical features may include extension beyond the prostate into adjacent tissues, the presence of septations or loculated cavities, and evidence of necrosis [3,4]. As prostate abscesses most commonly occur in the fifth and sixth decades of life, presentation in younger men is unusual [2].

Prostate abscesses most commonly form secondary to acute bacterial prostatitis [1]. A key risk factor for these conditions is diabetes mellitus; when poorly controlled, it increases susceptibility to urinary tract infections (UTIs) through altering urine pH, increasing urinary glucose, and, more generally, through immunosuppression [1,5]. Other immunocompromised states, such as HIV infection, end-stage renal disease, liver cirrhosis, and malignancy, further impair host immune defenses and increase susceptibility to infection [1,2]. Voiding dysfunction, whether due to neurological conditions or mechanical obstruction (e.g., urethral strictures), contributes to urinary stasis and retrograde bacterial flow into prostatic ducts, facilitating infection and potential abscess formation [2,6]. The use of long-term indwelling catheters or intermittent self-catheterization is also a well-recognized risk factor, given the increased likelihood of recurrent UTI [2]. Other genitourinary instrumentation, including prostate biopsy, brachytherapy, and cryotherapy, can also serve as a direct route for bacterial inoculation into prostatic tissue [2]. Early recognition and prompt management are essential to prevent progression to systemic infection, sepsis, or other serious complications.

In recent years, gram-negative bacteria have become the most predominant causative organisms (*Escherichia coli* and *Klebsiella pneumoniae*), with atypical organisms mainly being culprits in immunocompromised patients [2]. Before the widespread use of antibiotics, prostate abscesses were complications of sexually transmitted diseases and were commonly caused by *Neisseria gonorrhoeae* and *Chlamydia trachomatis* [2].

Diagnosis of prostatic abscesses is challenging due to a broad range of signs and symptoms that often contribute to delayed diagnosis and management [1,2]. Initial symptoms are often lower urinary tract symptoms, most commonly dysuria, hematuria, and increased urinary frequency [2]. Some patients may present with difficulty passing urine, urinary retention, or perineal discomfort, which may point toward prostatic involvement [1,2]. Systemic manifestations of the condition can include fevers, chills, myalgia, and lower back pain [1]. Patients may solely present with systemic signs of infection, thereby contributing to diagnostic uncertainty [2]. In such cases, a digital rectal examination is helpful to assess for prostate involvement, often demonstrated by gland tenderness and a palpable boggy mass [3,4]. Failure of antibiotic treatment for suspected lower UTI may suggest a missed prostatic abscess and often prompts exploration through imaging, which is normally the first point of diagnosis [1,3].

Imaging the prostate through techniques such as MRI, CT, or transrectal ultrasonography (TRUS) is imperative for diagnostic certainty [1]. While ultrasound is a convenient and often readily available bedside imaging modality, its user-dependent nature may mean there is variability in accurate diagnosis, leading to implications on subsequent management. This modality is especially useful when clinical suspicions of a prostate abscess are high and a patient is taken directly to the theater, as TRUS can guide intraoperative management [2]. Comparatively, if surgery is delayed, MRI is preferable as it offers more detailed imaging of anatomy and soft tissue characterization, including extraprostatic extension [1]. In contrast, although less specific, CT scanning is often more readily accessible out of hours and is useful when there is a broad differential diagnosis.

The mainstay of conservative management is broad-spectrum antibiotics and symptom addressment. This is frequently trialed as a first measure regardless of abscess severity. Antibiotic choice may vary by geographical location but aims to target genitourinary commensals and gut flora, including *Escherichia coli*, *Klebsiella*, and *Pseudomonas* species [3]. In instances where medical management fails, a surgical approach is often adopted as the next step. Emergency surgical intervention is considered at an earlier point for larger abscesses, typically those exceeding a 2 cm diameter [3]. Typical prostate abscesses have a higher chance of responding to antibiotic therapy alone, while atypical cases often have a comparatively poor response and require surgical intervention [1,7].

There are three main surgical approaches for prostate abscess drainage, namely, transurethral, transrectal, and transperineal. The use of ultrasound guidance intraoperatively offers surgeons clear visualization in real time to better assess anatomy and accurately locate the abscess during percutaneous aspiration, which can be done transperineally or transrectally [2]. Transperineal aspiration tends to be performed under general anaesthetic with the patient placed in the lithotomy position. TRUS is used to guide the surgeon so the needle punctures through the perineum and into the prostate abscess, allowing for aspiration [2]. Transrectal-guided aspiration is performed under local anesthesia with the patient in the left lateral decubitus position. TRUS is used to locate the abscess, and a needle is placed through the rectal wall into the prostate abscess for aspiration [2]. These methods are chosen for draining small prostatic abscesses (less than 2 cm diameter) and abscesses within the peripheral zone of the prostate [1,2]. Transrectal aspiration is often considered first line as it only requires local anesthesia (low anesthetic risk), has a lower risk of complications, and can be repeated if needed [1]. However, abscess recurrence is a major limitation of this method, particularly in the context of larger and multiloculated abscesses [7]. Recurrence rates after a single aspiration are 15-33% [1].

The transurethral approach (transurethral de-roofing) involves localizing the prostate abscess and resecting the prostatic tissue situated around the cavity’s neck. This opens up the abscess so any contents can drain into the urethra [3]. The transurethral approach is the preferred method for larger abscesses as it offers more effective drainage [7]. However, it has a greater scope for long-term postoperative complications, including urethral strictures, urinary incontinence, retrograde ejaculation, and may miss smaller abscesses [1,7].

The least commonly used surgical approach is open drainage via transperineal incision. There is limited literature surrounding this approach for atypical prostate abscesses, but it remains the least preferred method for typical prostate abscess drainage due to higher risks of fistula formation and infection [1]. It is generally only considered if the abscess has spread more extensively, affecting deeper tissues such as the levator ani muscle [3]. Ultimately, this method creates larger open wounds and involves a longer healing process compared to aspiration methods.

Wounds often heal through primary or secondary intention. Primary intention wound healing involves opposition of the exposed skin surfaces and may use deep and superficial sutures to hold tissue together [8]. In secondary intention, the wound is left open and heals without opposed skin edges [8]. Healing through primary intention enables faster healing and reduced risk of infection, whereas healing through secondary intention reduces the risk of abscess formation [9].

## Case presentation

A 45-year-old male with no significant past medical history presented to the Emergency Department with a three-day history of lower urinary tract symptoms of reduced urine output and dysuria, accompanied by lethargy and constipation. On examination, he was diaphoretic and tachycardic at 105 beats per minute. He also experienced rigors despite maintaining an afebrile state with a temperature ranging from 97°F to 99°F.

Initial laboratory investigations revealed elevated lactate and C-reactive protein (CRP) levels, stable renal function, and no other biochemical evidence of organ dysfunction (Table 1). Given his presenting symptoms, a bladder scan was performed, demonstrating a bladder volume of 750 mL. As this exceeded 500 mL, the patient was deemed to have urinary retention and was subsequently catheterized [10]. Following catheterization, his post-void residual volume was 0 mL, and he continued to produce sufficient amounts of urine as per his weight (greater than 40 mL/hour) [11]. A sample of catheterized urine was collected and sent for microscopy, culture, and sensitivity testing. Corresponding blood cultures were not obtained at this stage despite symptoms suggestive of infection; the reasoning for this remains unclear. A point-of-care urinalysis dip demonstrated blood but no other abnormalities.

**Table 1:** Blood test results.

Blood parameter (normal range)	Day from the first presentation to the hospital											
	0	1	2	3	4	5	6	7	8	9	10	11
White Cell Count (4.5–11.0 × 10^9^/L)	14.6	-	-	20.8	25.3	26.8	23.5	22.6	14	16	12.6	13.5
C-reactive protein (0–5 mg/dL)	187	-	-	195	229	340	282	242	89	51	26	14
Estimated glomerular filtration rate (>90 mL/minute/1.73m^2^)	>90	-	-	>90	>90	>90	>90	>90	87	>90	>90	>90
Lactate (0.0–1.0 mmol/L)	3.1	2	-	-	-	-	0.8	-	-	-	-	-
Capillary blood glucose (4.0–7.0 mmol/L)	6.7	5.2	-	-	-	-	5.4	-	-	-	-	6.4

Intravenous maintenance fluids were initiated, and empirical intravenous antibiotics (gentamicin) were administered to treat a suspected lower UTI. The intravenous route was selected in light of the patient’s persistent tachycardia, rigors, and elevated inflammatory markers of uncertain etiology. Despite these findings, he was not considered septic given his normotensive status, absence of fever, and no further signs of organ dysfunction.

**Figure 1:**
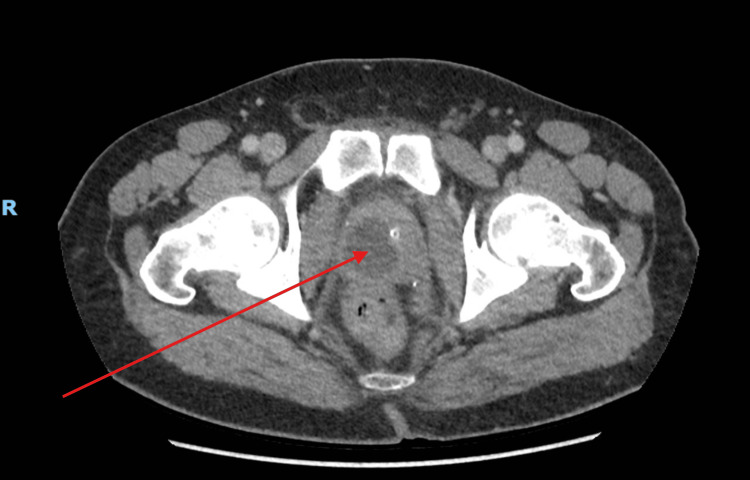
CT of the abdomen and pelvis on the day of admission (day zero). The red arrow points to the prostate abscess. The letter R depicts the right-hand side of the patient.

Regarding imaging, although ultrasonography may have been an appropriate initial modality, it was unavailable during out-of-hours service when the patient presented. To avoid diagnostic delay, a CT of the abdomen and pelvis was performed to assess for any intra-abdominal collections, bladder wall thickening, and genitourinary tract abnormalities. This unexpectedly revealed a low-attenuation prostatic lesion, most consistent with a prostatic abscess (Figure 1).

Urology consultation was sought urgently, and the team recommended hospital admission for continued intravenous antibiotic therapy and close monitoring. Despite being thoroughly counseled on the risks of incomplete management of a prostatic abscess, the patient elected to leave the hospital against medical advice. He was provided with comprehensive safety-netting instructions and scheduled for follow-up in the surgical triage unit in 12 hours’ time. Unfortunately, the patient did not attend the planned follow-up appointment, and the Urology team was unable to establish further contact.

The patient re-presented to the hospital two days later, directly to the surgical triage unit, as he had developed further symptoms and became increasingly unwell. He was now experiencing pain, swelling, and spreading erythema over his right buttock (adjacent to the anus, spreading laterally over 50% of the skin surface). On examination, the right buttock was indurated, erythematous, inflamed, and tender. No pus or discharge was visible. His tachycardia had progressed to 115 beats per minute, yet he remained afebrile and normotensive. His inflammatory markers had significantly worsened (Table 1), and coupled with his clinical deterioration, this reflected a progression to sepsis. He was admitted to the hospital under the Urology team. Intravenous co-amoxiclav and gentamicin were commenced. Unusually, his clinical observations remained stable at this time. General Surgery was consulted due to the newly reported symptoms in the buttocks. Their impression was gluteal cellulitis and an ischiorectal abscess secondary to the suspected prostate abscess.

Five days after the patient’s initial presentation to the hospital, and three days following re-presentation, the decision was made to proceed with surgical intervention. This escalation to operative management was prompted by a worsening biochemical profile (Table 1) despite three days of intravenous antibiotic therapy.

**Figure 2:**
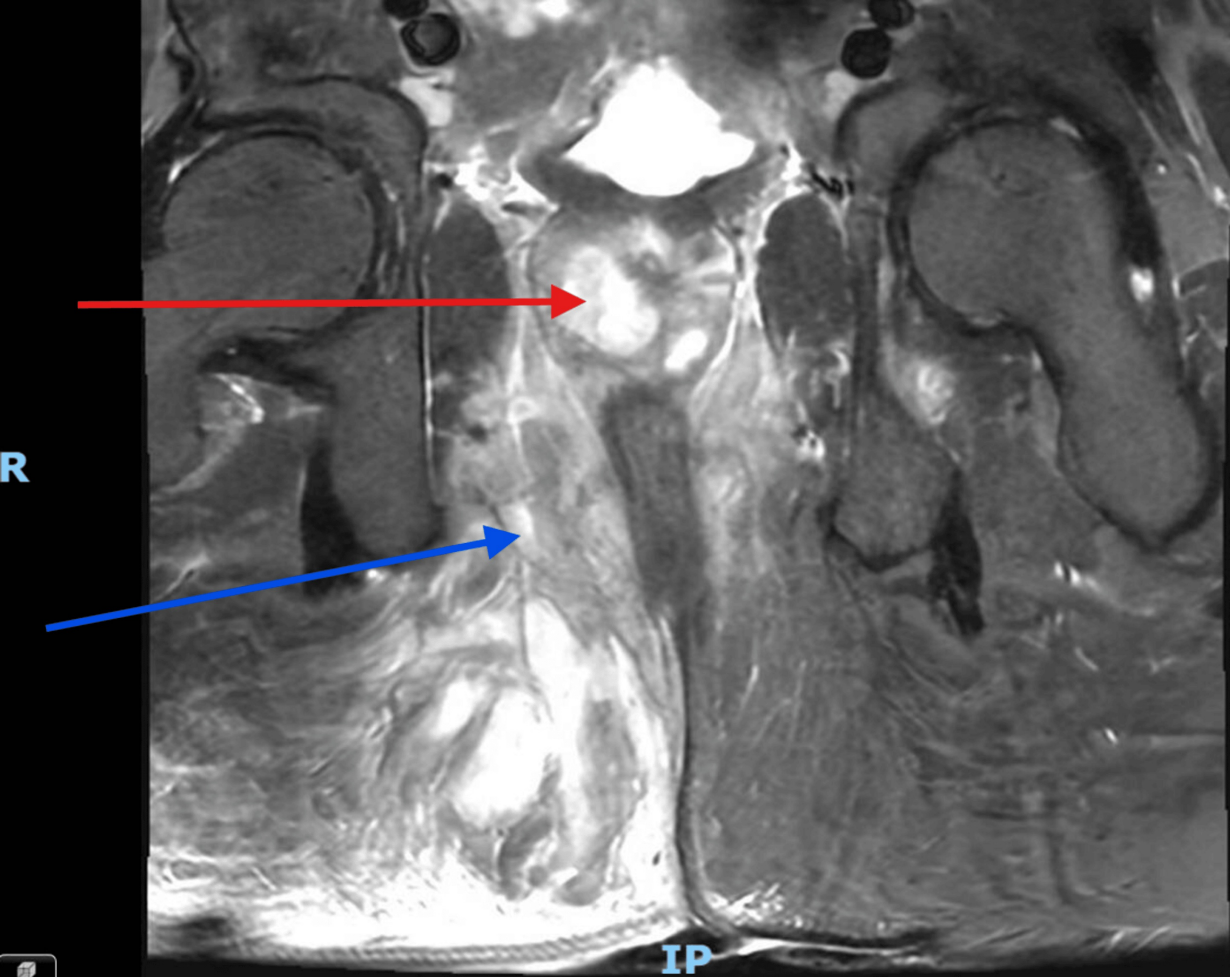
MRI of the prostate taken five days after the initial presentation to the hospital. The red arrow points to the prostate abscess within the prostate gland. The blue arrow points to surrounding tissue edema, including inflammation of the right ischiorectal fossa and gluteus maximus. The letter R depicts the right-hand side of the patient, and IP depicts the inferior-posterior of the patient.

**Figure 3:**
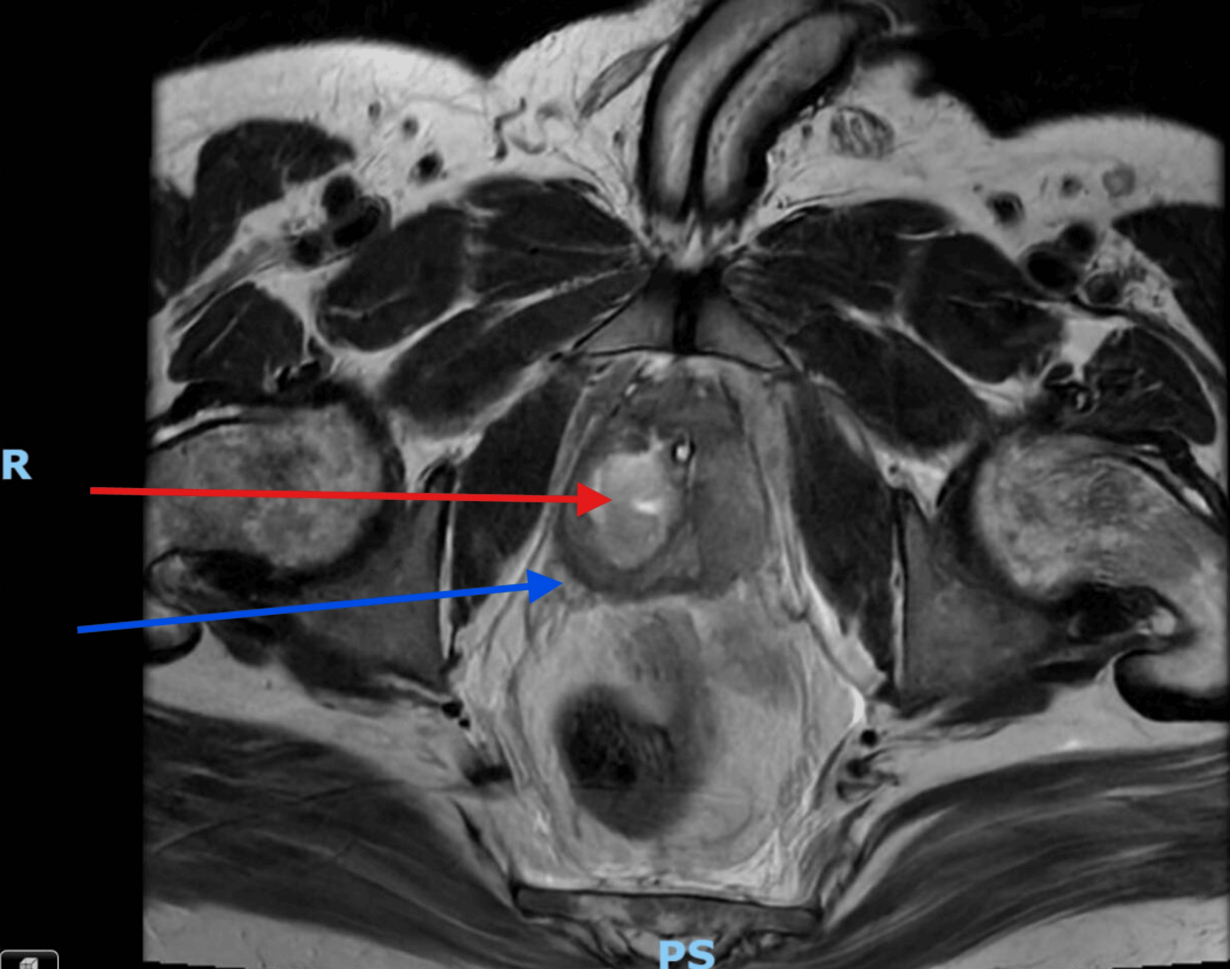
MRI of the prostate taken five days after the initial presentation to the hospital. The red arrow points to the prostate abscess within the prostate gland. The blue arrow points to extraprostatic extension of the prostate abscess. The letter R depicts the right-hand side of the patient, and PS depicts the posterior-superior of the patient.

On the day of surgery, a preoperative pelvic MRI was performed to guide operative planning. The imaging demonstrated a well-defined prostatic abscess with extension into the right ischiorectal fossa, associated with multiloculated collections in the gluteal region and edema involving the anal canal and levator ani muscles (Figure 2). Extension of the abscess into the ischiorectal fossa and its multiloculated nature signify that it was atypical (Figure 3). In contrast, typical prostate abscesses are generally confined to the prostate gland parenchyma and do not demonstrate compartmentalization or spreading infection to surrounding tissues [4].

Given the complexity of the abscess and its extension beyond the prostate, the procedure was jointly undertaken by Urology and General Surgery, as collections within the ischiorectal fossa are typically managed by the General Surgical team. The operation was performed under general anesthesia. Consultant surgeons examined under anesthesia and wide drainage of the prostatic abscess, extraprostatic extension, and associated collections.

An open transperineal (paramedian) incision was selected to access the abscess cavity, as this approach provided optimal exposure of the ischioanal fossa where the abscess extended. Upon incision, purulent material was evacuated onto the skin surface. Digital exploration of the abscess cavity was subsequently performed, extending laterally toward the ischioanal fossa, superiorly toward its roof, and superficially toward the base of the scrotum. This allowed expression of pus from all loculations within the abscess, resulting in complete drainage. The surrounding tissue was thoroughly debrided to ensure adequate source control and minimize the risk of necrosis and further infection. The wound was left open after the theater (no sutures), and the postoperative plan specified wound healing by secondary intention. This was deemed the safest approach for abscess healing to allow for continuous drainage, reduced reformation of pus, and minimized abscess recurrence compared to primary intention wound healing [9].

Postoperatively, the choice of intravenous antibiotics was switched to clindamycin and meropenem in line with the Antimicrobial Trust guidelines for skin and soft tissue infections. This ensured coverage for necrotizing fasciitis, although the patient did not exhibit clinical features of this despite intraoperative suspicions that this may arise. The risk of necrotizing fasciitis was heightened by the atypical nature of the prostate abscess and a delay in definitive management. Therefore, antimicrobial therapy was escalated as a prophylactic measure. Clinical and biochemical improvement was seen following the operation and with escalation of antibiotics; however, tissue cultures taken in the theater reported growth of multiple organisms (*Escherichia coli*, extended-spectrum beta-lactamase positive, *Staphylococcus epidermis*, *Enterococcus faecalis*, and *Staphylococcus aureus*). Histology showed scattered necrotic tissue but no dysplasia or invasive malignancy.

After surgery, the patient had an uneventful recovery with no noted postoperative complications. All clinical observations had now normalized, including the previous tachycardia. His catheter was removed, and he was able to pass good volumes of dilute urine independently. Intravenous antibiotic therapy continued for five days postoperatively (meropenem and clindamycin). Before discharge, his CRP had downtrended to 14, and his white cell count was 13.5 × 10^9^/L (Table 1). He was discharged six days after surgery and was stepped down to oral antibiotics (seven-day course of co-amoxiclav). This was to cover for superficial tissue infection in light of mildly raised inflammatory markers. In addition, he was advised to attend his General Practice for daily dressing changes in the community. Given previous non-compliance with medical care, the patient was provided with the contact information of the surgical triage unit and was advised to attend directly if needed.

Three weeks after discharge, the patient was reviewed in an outpatient Urology Clinic. The wound was observed to be healing well with no signs of superficial skin infection. There were no postoperative complications. During this follow-up, risk factors contributing to prostatic abscess formation were explored (biochemically, clinically, and through history-taking), as these had not been discussed during his hospital admission despite an immunodeficiency panel being sent off for laboratory analysis. Although his blood glucose levels were within the normal range throughout admission (Table 1), a glycated hemoglobin (HbA1c) test conducted on the day of discharge revealed an elevated value of 50 mmol/mol (reference range: <42 mmol/mol), suggesting the possibility of previously undiagnosed type 2 diabetes mellitus. This was discussed in clinic as opposed to during his acute hospital admission, as this biochemical data only became available after discharge. The patient reported no prior symptoms suggestive of diabetes, had no family history of the disease, and had no recorded episodes of hyperglycemia, which may explain why the condition had previously not been investigated. He subsequently made a full recovery from his acute urological illness. Ongoing evaluation, diagnosis, and management of possible diabetes mellitus were arranged through his primary care physician.

## Discussion

Vague initial symptoms and a broad differential for prostate abscesses generally lead to a delay in diagnosis and management [1,2]. There is a large overlap between UTI and prostatic abscess symptoms, often leading to initial misdiagnosis. Other differentials may include pyelonephritis, intra-abdominal collections, and prostatitis [2]. In this case, the initial working diagnosis was a lower UTI; however, the patient’s elevated inflammatory markers and signs of systemic involvement prompted further investigation with CT, which incidentally revealed a prostatic abscess. Had raised inflammatory markers not been apparent, there may have been a further delay in diagnosis with possible morbidity and mortality consequences. It is worth considering that most patients with prostatic symptoms first present to primary care, where blood testing may not be available; there may be a subsequent delay in diagnosis and management in secondary or tertiary care, where blood tests and imaging are performed.

Abscesses usually present with a swinging pyrexia; however, it is noteworthy that, in this case, the patient was afebrile throughout [12]. This offers a key learning point that not all prostate abscesses or infections demonstrate temperature changes; hence, it is important to review a patient holistically when assessing disease severity and ongoing management options. In this case, the stability of his temperature and blood pressure may have masked the systemic impact of the atypical abscess and the severity of associated infection. These findings could indicate adequate compensatory mechanisms in a young patient with optimal physiological reserves, but do not necessarily imply that the antibiotic therapy was effectively controlling the infection.

In this case, a delay in appropriate management was attributed to a combination of patient- and hospital-related factors. The patient was noncompliant with the initial medical advice provided, chose to self-discharge, and subsequently failed to return to the hospital within the recommended timeframe. This possibly contributed to further clinical deterioration, abscess enlargement, and the development of multidrug resistance in affected tissues, subsequently making the condition more difficult to manage conservatively. Ultimately, this necessitated escalation to operative intervention as a last resort to save the patient’s life. Upon reflection, a three-day delay in taking the patient to theatre after re-presentation, despite markedly elevated inflammatory markers, may have represented an inappropriate delay. Delayed management of prostatic abscesses has been associated with unfavorable outcomes, including sepsis and mortality [1]. Standard practice dictates that emergency surgical management should proceed promptly when conservative management fails, as evidenced in this case by persistently elevated inflammatory markers, ongoing sepsis, and clinical deterioration despite antibiotics [13].

Historically, the only method available for managing prostate abscesses was open drainage, as conservative and minimally invasive approaches were not feasible in an era without antibiotics or modern technological advancements [14]. Over time, such advancements have led to a marked decline in the use of open drainage [3]. However, it is noteworthy that in cases involving extraprostatic extension or multiloculation (atypical cases), the open approach continues to provide the most favorable outcomes [3]. This technique allows for thorough drainage and debridement of atypical abscesses through direct visualization of the abscess cavity. Aspiration or transurethral deroofing does not offer this direct visualization, making complete drainage of the atypical abscess challenging. As demonstrated in Figure 2 and Figure 3, the patient exhibited extensive inflammation and collections in the ischiorectal fossa and gluteal region as sequelae of the prostatic abscess. This made the open approach via a transperineal incision essential, as it permitted full access to and drainage of the associated collections and extraprostatic components of the abscess. The open technique also enabled thorough debridement of these areas, which demonstrated necrosis on histological analysis. Conversely, aspiration and transurethral deroofing approaches are superior in typical cases because they are minimally invasive, associated with fewer risks, and can achieve adequate drainage in unifocal, localized, and smaller abscesses (less than 2 cm diameter) [1]. It is important to note that open surgical techniques may not fall within the skill set of all urologists, potentially leading to a preference for aspiration or deroofing methods [7]. Well-recognized complications of open transperineal drainage include incontinence, impotence, wound dehiscence, and infection [1]. These significant considerations may deter surgeons from selecting this approach when other feasible options are available. Remarkably, there are no universally accepted guidelines for the medical or surgical management of prostatic abscesses [3].

Necrotizing fasciitis is a severe bacterial infection of the subcutaneous tissues associated with significant morbidity and mortality. It is characterized by the rapid spread of infection along fascial planes, resulting in extensive tissue destruction and necrosis [15]. Postoperatively, the patient was treated with intravenous meropenem and clindamycin in accordance with local guidelines for necrotizing fasciitis, as the operating surgeons considered the patient to be at high risk for developing this condition given the atypical complexity of the abscess and the intraoperative findings of extensive surrounding tissue involvement. In this case, the prostatic abscess had extended beyond the prostate and was surgically drained, creating the potential for bacterial spread into the periprostatic, perirenal, and scrotal fascial planes [16]. Although these tissues were thoroughly debrided, the risk of necrotizing fasciitis remained substantial due to possible bacterial seeding during extraprostatic debridement. Additionally, the delay in surgical intervention may have allowed for prolonged bacterial proliferation and deeper invasion into fascial layers, further predisposing to necrotizing fasciitis. Histology from intraoperative tissue samples confirmed the presence of necrotic cells, underscoring both the severity of infection and the appropriateness of postoperative antibiotic escalation. The patient’s preoperative clinical deterioration and worsening systemic symptoms further support the likelihood of progressive infection. Had blood cultures been obtained during admission, they would likely have demonstrated the growth of multidrug-resistant organisms similar to those identified in the intraoperative tissue cultures.

Despite the presence of a grossly atypical prostatic abscess, severe polymicrobial infection, and histological evidence of tissue necrosis, surgical management combined with the use of carefully selected broad-spectrum antibiotics resulted in successful treatment in this case. A follow-up outpatient clinic appointment a few weeks after discharge observed a well-healing wound through secondary intention and no signs of prostate abscess recurrence. Although the primary intention is typically preferred for the healing of iatrogenic external genitourinary wounds, there is evidence to suggest a higher postoperative risk of recurrence with this method compared to secondary intention healing [8]. This is important to consider when selecting a wound healing method. The success of secondary intention in our case suggests this may be the preferred method for prostate abscesses undergoing open drainage to minimize risks of recurrence.

Additionally, risk factors for prostate abscess formation were not identified or discussed during inpatient admission, but were subsequently reviewed during the outpatient follow-up. Results from an in-hospital immunodeficiency panel, including human immunodeficiency syndrome and hepatitis testing, were negative. Notably, the patient’s HbA1c level was elevated despite normal capillary blood glucose readings throughout admission (Table 1) and an absence of hyperglycemic symptoms. This finding may suggest underlying diabetes mellitus; however, according to the National Institute for Health and Care Excellence guidelines, an asymptomatic patient cannot be diagnosed based on a single elevated HbA1c result [17]. Consequently, confirmation testing and subsequent management were handed over to his primary care physician to facilitate continuity of care. It is possible that previously unidentified diabetes mellitus contributed to initial prostate abscess formation, given that it is an independent risk factor [1]. This highlights the importance of screening for risk factors in atypical presentations to support earlier identification and management of comorbidities, ultimately improving patient outcomes and reducing risks of recurrent prostate abscesses and associated complications.

## Conclusions

This case illustrates the challenges of delayed diagnosis and management of an atypical prostatic abscess in a young male with good baseline health. It highlights that clinical observations alone may not provide an accurate picture of abscess severity and associated complications, so they should not be relied upon in isolation to reflect the patient’s clinical picture. Open drainage via a transperineal approach successfully managed this atypical abscess, which was multiloculated with extraprostatic extension. This technique uniquely allows direct visualization and debridement of necrotic tissue, which can be particularly beneficial following antibiotic failure in the presence of multidrug-resistant organisms or suspected necrotizing fasciitis. Although open surgery carries inherent risks, it may provide superior outcomes in carefully selected patients where alternative approaches are unlikely to achieve complete drainage. This case demonstrates that transperineal open drainage can be a safe and effective option, enabling successful source control and recovery without abscess recurrence. Despite operative intervention, the added use of guideline-directed antibiotics in atypical cases is paramount as it jointly facilitates source control, management of sepsis, and reduces the risk of infection-related complications postoperatively. Identification and addressment of risk factors for prostatic abscesses at the earliest opportunity may prevent their occurrence in the first instance, which would ultimately negate all associated risks. Future considerations include the need for standardized guidelines to assist clinicians in the timely diagnosis, management, and follow-up of atypical prostatic abscesses. Developing and maintaining open transperineal drainage skills among urologists may enhance the management of this uncommon but serious condition, ultimately improving morbidity and mortality outcomes in the long term.
